# A Master of Public Health with a Concentration in Mass Gatherings Health

**DOI:** 10.7759/cureus.5944

**Published:** 2019-10-19

**Authors:** Mohammad Hasan Rajab

**Affiliations:** 1 Biostatistics, Epidemiology, and Public Health, College of Medicine, Alfaisal Univerity, Riyadh, SAU

**Keywords:** master of public health (mph), mass gatherings health, hajj, umrah, ziarah, alfaisal university, kingdom of saudi arabia, graduate academic program

## Abstract

Introduction

The Kingdom of Saudi Arabia (KSA) is in a unique position and different from other nations that periodically host an event, such as the World Cup or the Olympics. The KSA is faced annually with millions of pilgrims coming from around the world to participate in the Hajj, Umrah, and Ziarah rituals. Continuously hosting such huge mass gatherings poses overwhelming challenges to the Saudi healthcare system. Consequently, an additional specialized cadre of qualified public health personnel is needed. The purpose of this article is to describe the establishment of an academic public health program that addresses this need.

Materials & Methods

In response to the dire need for public health specialists, a landmark project was launched in 2013 at Alfaisal University (AU). The project aimed to establish a Master of Public Health (MPH) program with a concentration in the health aspects of mass gatherings. The MPH program was created in collaboration with the United States-based Partners HealthCare International (PHI), the Saudi Global Center for Mass Gatherings Medicine (GCMGM) at the Ministry of Health (MOH), the Ministry of Education (MOE), and the Ministry of Hajj and Umrah (MOHU). A needs assessment was conducted to help in the program development. The needs assessment drew on the unpublished findings of an institutional review board (IRB)-approved anonymous market survey that was administered to prospective students of the MPH program.

Results

The needs assessment study revealed the need for an MPH program with concentrations in mass gatherings health, health policy and management, and biostatistics and epidemiology. Consequently, we designed an MPH program to include these three concentrations. In particular, the mass gatherings health concentration aimed at preparing qualified public health specialists to help manage risks to the Saudi public health system, to analyze health data, and to recommend policies associated with the continuous mass gatherings events. Challenges to program development included working with multiple governmental agencies, absence of a frame of reference to develop a program curriculum, a lack of qualified faculty, lengthy and tedious government approval procedures, and inadequate funding. After obtaining the required approvals, the MPH program with a concentration in mass gatherings health was inaugurated in the fall of 2016. This program is accredited by the Saudi government to prepare public health specialists trained and locally certified in mass gatherings health issues.

Conclusions

The public health experts of the AU have established what may be the first MPH program with a concentration in mass gatherings health. The main challenges to program development were identified, and appropriate actions to meet these challenges were taken. This innovative MPH produces certified specialists equipped with the analytical and policy-making tools necessary to continuously monitor and improve the public health aspects of mass gatherings. Besides, the program provides a frame of reference for the development of unique public health programs, not only in the KSA but worldwide.

## Introduction

The Kingdom of Saudi Arabia (KSA) has extensive experience managing the yearly tradition of hosting millions of Muslim pilgrims from all over the world to the Two Holy Mosques: The Sacred Mosque in Mecca for Hajj and Umrah and the Prophet’s Mosque in Medina for Ziarah. These events fit the World Health Organization’s (WHO) definition of mass gatherings [1]. It is characterized by the concentration of people at a specific locality for a particular purpose over a determined period which may strain the available resources of the hosting community. These rituals take from a few hours to a few days to complete, and two of them, Umrah and Ziarah, have a distinct characteristic of being nonstop for the whole year. Thus, the tasks of caring for the pilgrims and protecting the local public are continuous processes with a steady stream of ongoing pilgrims interspersed with over two million persons during the peak of Hajj [2].

Hajj, the pilgrimage to the city of Mecca, is one of the five pillars of Islam. Muslims worldwide who can afford to perform Hajj and who are healthy are required to complete it once at some point during their lifetime [3]. Mecca (Makkah) is in Western Saudi Arabia, inland from the Red Sea coast. At the center of Mecca is the Kaaba, a stone building in the center of the Sacred Mosque, around which the pilgrims conduct some of the rituals of Hajj. Hajj must be undertaken during specific dates according to the Islamic lunar calendar. In 2019, according to the website of the Ministry of Hajj and Umrah (MOHU), the total number of Hajj pilgrims was 2,489,406. Of those, 44% were females, and about 26% came from within the KSA [2].

Umrah is the second type of religious ritual that Muslim pilgrims are expected to perform in their travels to Mecca. Umrah is a shorter version of Hajj, which may only require a few hours to complete. However, greater flexibility is permitted by this ritual, given that it is an optional pilgrimage that may be performed at any time of the year. According to the MOHU, the total number of Umrah visas issued in 2019 during the first 10 days of the month of Ramadan was 7,222,369 [2].

Most Hajj or Umrah pilgrims travel to Medina, the city of the prophet, to perform the Ziarah ritual, which is a visit to the prophet’s mosque. Medina is located northwest of Mecca, inland from the Red Sea coast as well. At the center of the city is the Prophet’s Mosque. The Ziarah ritual can be completed in a few hours; however, most pilgrims choose to stay in Medina for a few days afterward.

The significance of the three rituals to the KSA is not only religious but symbolic as well. These rituals place the KSA at the heart of the Muslim World. Pilgrims of all ages, who speak different languages, possess different levels of education, and exhibit varying health conditions continuously travel from close to 200 countries to perform these rituals [2]. 

Saudi Arabia has mostly a desert climate that is very hot, dry, and dusty. These conditions, combined with the risk of injuries and the potential physical exhaustion during and after performing the mass gatherings rituals, increase the health risks of the pilgrims, including disease transmission [4-5]. Therefore, caring for and protecting the pilgrims and the local population are neverending and enormously challenging tasks. To that end, the KSA enforces medical requirements as part of the visa application process for the three rituals. These requirements include proof of vaccination for diseases, such as seasonal influenza, H1N1 (swine flu), and meningitis. Several medical facilities located in and around the holy sites provide free medical services to the pilgrims [6].

Over the years, the KSA has developed outstanding public healthcare programs, including immunization, environmental sanitation, and mobile health centers. These programs are only operational during the peak periods of the pilgrims’ travel. However, significant challenges posed by continuous mass gatherings events place exceptional strain on the Saudi healthcare system [7]. Consequently, the ability to identify, examine, and respond to a public health problem is potentially weakened. Examples of challenges posed by continuous mass gatherings include providing a safe environment for pilgrims, prevention of the spread of infectious diseases, the security and sufficiency of clean water, food, and sanitation for the pilgrims, and the provision of healthcare to pilgrims at the sites, especially those with chronic diseases [8].

In 2017, more than 90 million migrants were living in the European region [9]. Such massive population displacement and migration resulted in the loss of thousands of lives [9]. Moreover, this massive displacement and eventual cross-border gatherings, coupled with limited access to healthcare and healthcare specialists, may potentially result in the emergence and spread of infectious diseases [9]. This regional population displacement further highlights the need for local specialists who are experienced in aspects of public health issues of mass gatherings.

The latest expansion of the Two Holy Mosques has led to a sharp increase in the number of foreign Umrah visitors [10]. Also, the Vision 2030 plans to increase the number of pilgrims to 30 million. Consequently, there has been an increasing demand for qualified public health professionals to support the increase in the number of Umrah visitors and the Hajj and Umrah Vision Realization Program [10].

## Materials and methods

Following the university’s institutional review board (IRB) approval, this study was conducted to describe the establishment of an MPH program with a concentration in mass gatherings health. Located in Riyadh, Saudi Arabia, Alfaisal University (AU) is a private, non-profit, multicultural university with over 3,000 students representing over 40 nations [11].

Each year, several million pilgrims from all over the world come to the KSA to perform Hajj, Umrah, or Ziarah rituals. Hence, there is a demand for qualified public health specialists who observe health events, trends, and concerns to ensure quality public health programs for the protection of the pilgrims, as well as the health of the Kingdom’s inhabitants. In response to this demand, a landmark academic project was launched in 2013 at AU to establish an MPH program with a concentration in mass gatherings health.

This innovative academic program was established in close collaboration with the United States-based Partners HealthCare International (PHI). The MPH program was later launched with the support of the Global Center for Mass Gatherings Medicine (GCMGM) at the Ministry of Health (MOH), the Ministry of Education (MOE), the MOHU, and the WHO. The involvement of these entities, especially the Saudi government, is vital for success. This new MPH program was designed to produce specialists equipped with the analytical and policy-making tools necessary to continuously monitor and improve the public health aspects of mass gatherings.

Establishing a graduate program, especially in public health, can be a complicated and challenging task. It entails comprehensive planning and coordination [12]. To develop this MPH program, we completed the following tasks:

1. Draft the initial proposal 

2. Conduct a needs assessment

3. Team building 

4. Prepare and officially submit the program application to the MOE

5. Acquisition of required approvals to start the program

6. Develop a plan for the admission process

7. Program initiation and follow-up 

Program development began in 2013 with an idea proposed in writing by this study author to the Dean of the College of Medicine (COM) at AU. After proposal approval, a needs assessment was conducted to find out if there was a local need for an MPH program concentrated on the health aspects of mass gatherings. The assessment was done, in part, using reports, findings, and information available at the WHO, the Saudi MOE, MOH, MOHU, and the United States Center for Disease Control and Prevention’s website [6]. The needs assessment also drew on the unpublished findings of an IRB-approved anonymous market survey conducted by this study author. The market survey was administered to prospective students of the MPH program. The survey aimed to evaluate the local community’s need for a graduate program in public health with a concentration in mass gatherings health.

## Results

In conducting the needs assessment, the results of the market survey supplemented other sources of information, including our observations and experiences and guided our actions. The findings of the market survey are presented in Table 1.

**Table 1:** Results of a Market Survey Among Prospective Master of Public Health (MPH) Students of Alfaisal University

Characteristic	n (%)
Number of participants	85 (100.0)
Gender	
Male	52 (61.2)
Female	33 (38.8)
Citizenship	
Saudi	19 (22.4)
Non-Saudi	66 (77.6)
Age group	
18 - 24	65 (76.5)
25 - 34	13 (15.3)
35+	7 (8.2)
Education	
Some college	61 (71.8)
College graduate	16 (18.8)
Post-graduate	8 (9.4)
Current job title	
Medical student	65 (76.5)
Physician/intern	12 (14.1)
College faculty/staff	5 (5.9)
Researcher	2 (2.4)
Nurse	1 (1.1)
Interested in the MPH	
Yes	67 (78.8)
Preferred MPH concentration	
Health policy and management	42 (49.4)
Mass gatherings health	22 (25.9)
Biostatistics and epidemiology	21 (24.7)

Of the market survey participants, 38.8% were females, 22.4% were Saudi citizens, and 76.5% were medical students. The ages of 76.5% of the participants ranged from 18 - 24 years. Moreover, 78.8% of the participants reported interest in the proposed MPH program. Most of the respondents preferred the health policy and management concentration, and a quarter was interested in the mass gatherings health concentration.

Moreover, up until a few years ago, only a small number of specialists worked in the Kingdom’s public health sector. In 2014, for example, only 3,704 public health specialists were working for the MOH [13]. Despite the availability of about 30 medical colleges in the KSA that concentrate on curative medicine, only a few offer public health programs; none has a concentration in mass gatherings health. Consequently, the number of eligible school graduates and medical professionals who wish to study public health appear to be higher than available seats. Thus, the Saudi government enabled Saudi students to attend prominent Western institutions of public health, such as Emory University and Rollins School of Public Health [14].

Once the need for such an MPH program was established, the next stage was team building. An extensive search was conducted for national and international public health experts with experience in mass gatherings health and related areas. Since 2008, AU has an ongoing affiliation with Partners Harvard Medical International (PHMI), now called Partners HealthCare International (PHI), for the establishment of a state-of-the-art COM.

During the COM program development, the PHI joined the AU leadership and faculty in the medical program’s strategic planning and implementation. These activities included faculty and curriculum development, assessment of students, faculty, and program, as well as advice on infrastructure and governance. PHI experts also participated in similar oversight and advisory roles in establishing the MPH Program. In January of 2017, the GCMGM collaborated with the WHO to lead a global consultation on risk assessment tools for mass gatherings health in Jeddah, Saudi Arabia. The newly established MPH team and PHI were invited to participate and interact with world experts on this topic [5].

The next stage was to complete and submit a program approval form to the MOE. The form included sections on program justification, aims, curriculum development, admission requirements, description and justification of new courses that must be created, and a reference to the student manual that would be created. Once the form was completed, it was submitted for approvals by the COM Board, the University Board, and finally, by the MOE.

The next stage was the acquisition of the required approvals of the new program. Obtaining approvals for a new graduate degree program in the KSA includes several stages. First, new degree programs require college and university board reviews and approvals. The second stage is to submit for MOE review, including evaluation by an external independent panel of subject experts - usually, another Saudi university that has experience in this area. Finally, apply for approval by the MOE. However, the external review process was the most demanding and time-consuming stage.

This newly created MPH program adopted an innovative curriculum designed to serve the public health needs of the KSA. It provides quality education to English-speaking graduate students in the KSA and beyond. The program requires a minimum of 43 credits to be completed in two years, i.e., four semesters, with a maximum of 12 hours per semester. It includes a practicum followed by a thesis. Students can choose one of three tracks: mass gatherings health, healthcare policy and management, or biostatistics and epidemiology. In particular, the mass gatherings health track provides strong public health education and specialized training in mass gatherings health issues. It was quite a challenging task to develop a curriculum for this MPH program without a credible frame of reference. The principal consideration of the newly established curriculum was that the Saudi public health workforce requires intense preparation in analytical methods and research techniques.

Next, the MPH team focused on the development of an admission process. Admission requirements included a baccalaureate degree from a regionally accredited college or university, a minimum undergraduate grade point average of 3.00 on a 4.00 scale, the Test of English as a Foreign Language (TOEFL®) (Educational Testing Service, Princeton, NJ, USA) score of 500; and successful completion of an in-person interview with the program director or his designee. Students who do not meet some of these admission criteria are required to complete selected courses tailored towards improving English language proficiency or computer skills.

During the MPH program development, the MPH team faced many challenges. These challenges included the absence of a frame of reference to develop program curriculum, a lack of qualified faculty, lengthy and tedious program approval procedures, limited graduates’ certification, and an inadequate number of scholarships for MPH students. Another encounter that we faced was a short time to plan the recruitment process properly. Only a few weeks before the scheduled starting date, we received the official program approval. As a result, insufficient time was available for proper planning and advertising.

Additionally, the word was out that some of the government funding programs had expired. Consequently, the first cohort consisted of only four self-paid students. The main challenges of the program development and implemented actions to meet these challenges are presented in Table 2.

**Table 2:** Main Challenges to Program Development and Implemented Actions AU: Alfaisal University; GCMGM: Global Center for Mass Gatherings Medicine; MOH: Ministry of Health; MOHU: Ministry of Hajj and Umrah; MPH: Master of Public Health; PHI: Partners HealthCare International; WHO: World Health Organization

Challenge	Actions
Absence of a frame of reference to develop program curriculum. While multiple institutions offer programs in humanitarian assistance in crisis situations, our search resulted in no MPH program worldwide with a concentration on mass gatherings health	An online search was conducted for national and international experts with experience in mass gatherings health; some agreed to join the MPH team at Alfaisal University. Besides, we collaborated with the experts of the MOH, GCMGM, PHI, and WHO
The local accreditation authority, known as the Saudi Commission for Health Specialties (SCFHS), certifies only medical professional graduates, i.e., medical doctors, dentists, and nurses	We continue to work with the SCFHS to expand the certification process to a full range of professionals, including those in the areas of healthy nutrition and health communication
An insufficient number of qualified faculty and public health specialists	We sought the support of experts from AU, PHI, WHO, GCMGM, MOH, and MOHU
Working with multiple governmental agencies and a lengthy graduate program approval process	We were successful in convincing the local authority to shorten the process by utilizing qualified international experts to review program applications
Program funding and an insufficient number of scholarships for MPH students	We partnered with the MOH to sponsor applicants to the newly established MPH program
Inadequate time to plan for student recruitment	We utilized social media to spread the word about the program. However, these extra efforts were not very successful.

After the main challenges to program development were identified, the MPH team took appropriate actions to meet these challenges and receive the MOE program approval. The first mass gatherings health cohort was launched in the fall of 2016.

## Discussion

Currently, the KSA offers the first MPH program with a concentration in mass gatherings health at AU. The immense need for public health specialists and the current scarcity of public health professionals underscored the rationale behind this newly created MPH program. This MPH program increases the capacity of the Saudi public health sector and prepares public health leaders to address some of the Kingdom’s mass gatherings health challenges of the 21st century. 

During the MPH program development, the MPH team faced many challenges, most of which were alleviated with the support of PHI and other governmental agencies. For example, the team was successful in convincing the local authority to shorten the program approval process by utilizing qualified international experts to review the program application. Also, the MPH team partnered with the MOH to sponsor applicants to the newly established MPH program. To deal with the issue of the lack of qualified faculty, the team collaborated with experts of the MOH, GCMGM, PHI, and WHO to help start the program. Moreover, we continue to work with the SCFHS to extend the certification process to include a full range of professionals, including those in the areas of healthy nutrition and health communication.

In recognition for and support of the newly established MPH program, the MOH, represented by GCMGM and in collaboration with AU, organized the Third International Conference on Mass Gatherings Medicine. The conference was under the theme “From Mass Gatherings Medicine to Mass Gatherings Health: Evolving Perceptions and Practices” [15]. The conference aimed to raise awareness of the significance of health aspects during mass gatherings events, including preparation and health response. Moreover, to further support this new program, the MOH signed an agreement with AU to sponsor a minimum of five MPH applicants annually.

The new MPH program was constructed with an eye on the future. We planned to perform periodic evaluations of the program curriculum conducted by students, peers, and external experts. Also, we planned to conduct continuous improvement of the curriculum, including the flexibility to add new concentrations. The program was designed to attract strong candidates and train them to be recruited after graduation by local, regional, and international academic and public health organizations. These organizations are aware of the role that these qualified public health professionals play in maintaining and improving the public health sector and becoming the future leaders of the field. Creating a new generation of public health practitioners and scholars with expertise in mass gatherings health empowers the position of the KSA as a global leader in mass gatherings health, one of the main goals of the Saudi Vision 2030 [10].

Finally, the expertise gleaned from this program might benefit international organizations as they share challenges and solutions, such as the United States Federal Emergency Management Agency (FEMA). While other experts involved in mass gatherings usually work with planned events that occur on a scheduled basis every two to 10 years, such as the Olympics, World Cup, and Kumbh Mela, the experience in KSA is a continuous flow of pilgrims all year long with a peak at the period of Hajj.

Some limitations that might have impacted the needs assessment should be addressed. For example, the sample size for the market survey was small. Besides, the sample was chosen conveniently from prospective students of the MPH program. Consequently, this may impact the generalizability of survey results.

## Conclusions

The KSA is in a unique position as different from other nations that periodically host an event such as the World Cup or the Olympics KSA. The KSA is faced annually with millions of pilgrims coming from around the world to participate in the Hajj, Umrah, and Ziarah. In collaboration with several Saudi governmental agencies, the PHI, and the WHO, the public health experts of AU have established what may be the first MPH program with a concentration in mass gatherings health. The program was designed to provide the Saudi healthcare system with qualified public health professionals to cope with the recent expansion of the Two Holy Mosques. Besides, it empowers the position of the KSA as an international leader in mass gatherings health.

The main challenges for program development included working with multiple governmental agencies, an insufficient number of faculty, absence of a frame of reference to develop program curriculum, and funding. Appropriate actions to meet the challenges that were taken included collaboration with the experts of the MOH, GCMGM, PHI, and WHO, as well as partnering with the MOH to sponsor applicants to the newly established MPH program. This innovative MPH program provides a frame of reference for the development of unique public health programs not only in the KSA but worldwide.
